# Dose-response relationship of core-specific sensorimotor interventions in healthy, well-trained participants: study protocol for a (MiSpEx) randomized controlled trial

**DOI:** 10.1186/s13063-018-2799-9

**Published:** 2018-08-06

**Authors:** Juliane Mueller, Josefine Stoll, Steffen Mueller, Frank Mayer

**Affiliations:** 1 University Outpatient Clinic, Sports Medicine and Sports Orthopaedics, University of Potsdam, Am Neuen Palais 10, House 12, 14469 Potsdam, Germany; 2 Professorship for Physiotherapy: Exercise Science and Applied Biomechanics, Trier University of Applied Sciences, Trier, Germany

**Keywords:** Sensorimotor training, Perturbation, Exercise, MiSpEx

## Abstract

**Background:**

Core-specific sensorimotor exercises are proven to enhance neuromuscular activity of the trunk, improve athletic performance and prevent back pain. However, the dose-response relationship and, therefore, the dose required to improve trunk function is still under debate. The purpose of the present trial will be to compare four different intervention strategies of sensorimotor exercises that will result in improved trunk function.

**Methods/design:**

A single-blind, four-armed, randomized controlled trial with a 3-week (home-based) intervention phase and two measurement days pre and post intervention (M1/M2) is designed. Experimental procedures on both measurement days will include evaluation of maximum isokinetic and isometric trunk strength (extension/flexion, rotation) including perturbations, as well as neuromuscular trunk activity while performing strength testing. The primary outcome is trunk strength (peak torque). Neuromuscular activity (amplitude, latencies as a response to perturbation) serves as secondary outcome.

The control group will perform a standardized exercise program of four sensorimotor exercises (three sets of 10 repetitions) in each of six training sessions (30 min duration) over 3 weeks. The intervention groups’ programs differ in the number of exercises, sets per exercise and, therefore, overall training amount (group I: six sessions, three exercises, two sets; group II: six sessions, two exercises, two sets; group III: six sessions, one exercise, three sets). The intervention programs of groups I, II and III include additional perturbations for all exercises to increase both the difficulty and the efficacy of the exercises performed. Statistical analysis will be performed after examining the underlying assumptions for parametric and non-parametric testing.

**Discussion:**

The results of the study will be clinically relevant, not only for researchers but also for (sports) therapists, physicians, coaches, athletes and the general population who have the aim of improving trunk function.

**Trial registration:**

German Clinical Trials Register, ID: DRKS00012917. Registered on 22 August 2017.

**Electronic supplementary material:**

The online version of this article (10.1186/s13063-018-2799-9) contains supplementary material, which is available to authorized users.

## Background

One main function of the trunk muscles is the compensation of external forces and unexpected loads in order to ensure stability of the body. Consequently, compensation of external loads ensures the stability and performance of the body during daily life as well as in dynamic, high-intensity activities [1–3]. Trunk stability, led by neuromuscular activity, is required to control trunk motion during repetitive (dynamic) loading situations and to avoid overloading. Importantly, reduced trunk stabilization, mainly characterized by an increased muscle response time, higher co-contraction and an altered muscle activity pattern, could contribute to overload and injury [4, 5]. Consequently, inadequate neuromuscular control during dynamic loading is discussed as an explanatory model in back pain etiology [6, 7]. Therefore, great emphasis has been placed on the importance of trunk stability, especially in situations requiring compensation of (unexpected) high loading induced, e.g., by perturbations [5, 8]. Potential factors underlying back pain have been discussed, including repetitive micro traumas and insufficiency of the muscle-tendon complex based on inadequate postural and neuromuscular control, reduced maximum trunk-strength capacity and trunk-muscle fatigue during dynamic loading [9]. Hence, optimizing neuromuscular core stability is considered beneficial for protection against sudden, repetitive and excessive overloading of the trunk [2–4, 8].

Exercise interventions are proven to improve trunk stability [10–12]. Strengthening exercises have been applied and shown to be effective. Moreover, alongside the focus on strengthening exercises, additional training methods preferring neuromuscular, sensorimotor training or combinations thereof have been shown to be effective, too [10–12]. However, the dose-response relationship between sensorimotor training using exercises under unstable and/or perturbed conditions and trunk function still remains unclear. Especially for sensorimotor training, the benefits in terms of maximum eccentric and concentric trunk strength and peak torque as well as neuromuscular activity adaptation in sudden, dynamic trunk-loading situations have not been systematically highlighted.

In a pilot feasibility study (unpublished material, RCT (MiSpEx), German Clinical Trials Register No. DRKS00004977) including *n* = 750 participants, it could be shown that a core-specific sensorimotor exercise program (four exercises; nine sessions over 3 weeks; three sets of 10 repetitions per exercise) led to a significant increase in trunk function (strength gain) and pain reduction compared to a control group (no intervention). A compliance analysis revealed that more than one session per week (at least six sessions over 3 weeks) were necessary to achieve significant enhancement (function/pain reduction). In a recent Cochrane review on motor control exercises for non-specific low-back pain, Saragiotto et al. [10] summarized that the exercise programs’ duration ranged between 20 days and 12 weeks, with a number of sessions ranging from one to five per week. However, the exact determination of a valid dose-response relationship describing a reduced exercise amount and intensity necessary to improve trunk function remains unclear. Hence, the purpose of this study is to explore and compare the effect of three different 3-week (home-based) sensorimotor training programs in comparison to an evident control intervention, each varying the amount and intensity of training. Outcomes are maximum isokinetic trunk strength and trunk response (neuromuscular activity) to sudden, high-intensity trunk loading in healthy, normal, active adults.

## Methods/design

### Study design and flow

**Fig. 1:**
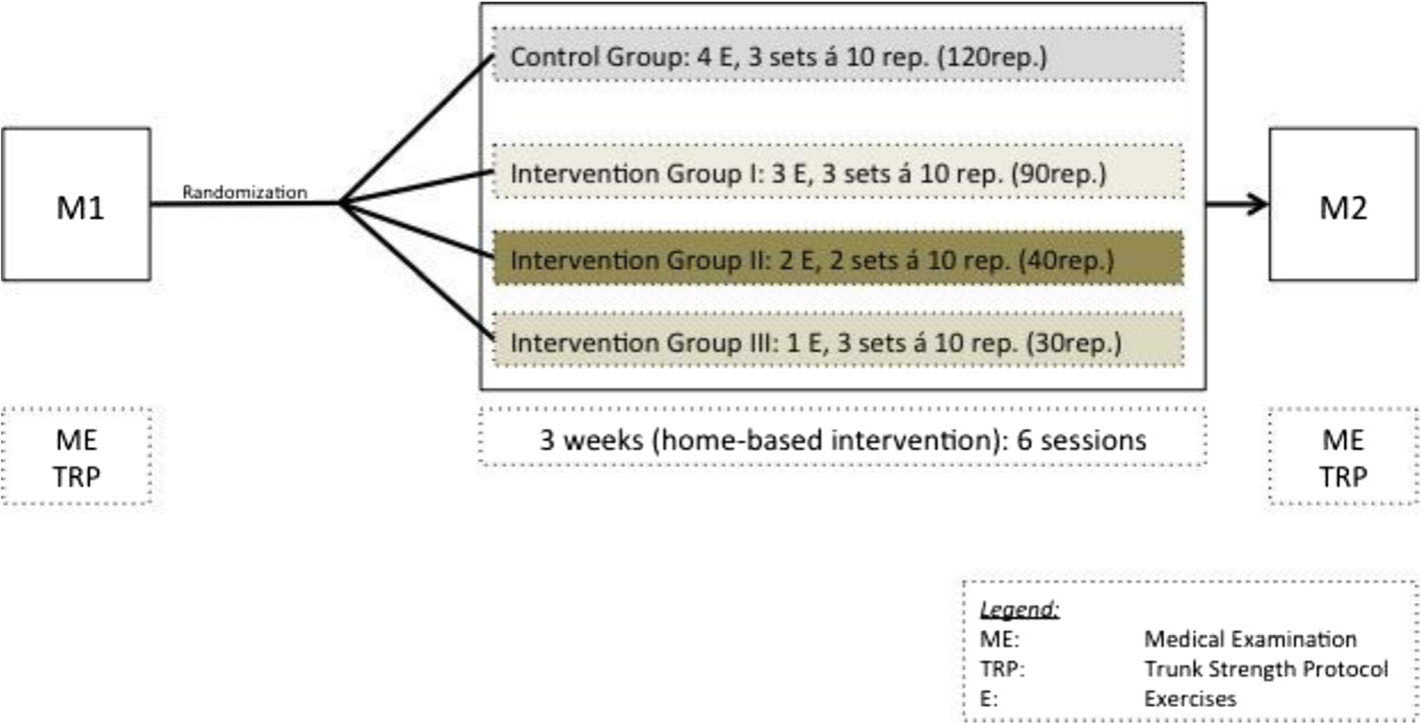
Trial flowchart

**Fig. 2:**
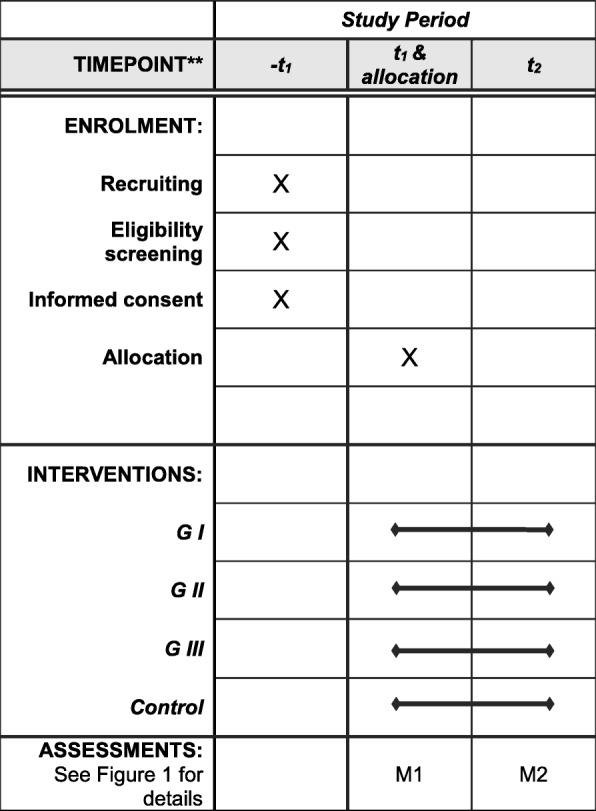
Standard Protocol Items: Recommendations for Interventional Trials (SPIRIT) Figure: schedule of enrollment, interventions and assessments

The study is designed as a single-blind, four-armed, randomized controlled trial with two identical measurement days (M1/M2), in which participants are allocated to either the experimental groups or the control group. All groups, interventions as well as control, receive 3 weeks of core-specific sensorimotor training organized in different home-based protocols. Potential participants are screened and examined by a sports medicine physician before baseline assessment and randomization to the groups to determine eligibility. Participants’ maximum trunk strength and trunk response to sudden, high-intensity trunk loading is assessed at baseline and after the 3-week intervention phase (Figs. 1 and 2; Additional file 1). All adverse events will be monitored and reported.

### Participants

#### Recruitment, screening and informed consent

Healthy, well-trained participants are recruited via the university outpatient clinic (e.g., athletes receiving annual health check-ups), flyers (displayed at the university and sports facilities) and existing contacts with training groups at the Olympic Center. Inclusion criteria are defined as age between 18 and 50 years and at least weekly training. Acute infection, pregnancy, any illness that contraindicates exercise, as well as acute or chronic back pain serve as exclusion criteria. Before voluntary participation, all subjects read and sign their written informed consent. The University’s Ethics Commission has approved the study (No. 23/2017).

### Intervention

#### Therapy flow

Trained and experienced physio- or sports therapists instruct the participants on all training programs (intervention and control; home-based). Following the randomization, both intervention and control group participants are scheduled for a guided training session, followed by a 3-week, home-based individual training.

#### Intervention groups and control group

Control and intervention group participants are instructed to perform six training sessions during the 3-week, home-based training phase. However, the number of exercises and sets per exercise vary between the control group and all intervention groups. Training frequency and number of exercises per group are detailed in Table 1.

**Table 1:** Intervention and control groups: details of training frequency and number of exercises per group

Group	Intervention	Total amount of sessions	Total time per session	Number of exercises	Sets per exercise
Control group C	MiSpEx protocol	6	30 min	4 (rowing, side plank, bird dog, standing balance)	3
Intervention group I G I	Better than nothing	6	20 min	3 (rowing, side plank, bird dog)	2
Intervention group II G II	Half-way done	6	10 min	2 (side plank, bird dog)	2
Intervention group III G III	Only once	6	5 min	1 (side plank)	3

The control group performs a standard protocol consisting of four different sensorimotor core-specific exercises (Table 2). All four exercises will be performed with three sets of 10 repetitions each, with a minimum of 30 s rest in between sets (unpublished material, RCT (MiSpEx), German Clinical Trials Register No. DRKS00004977, [13, 14]). In between exercises, a 2-min break (self-controlled) is allowed. The exercises are commonly described as: (1) bird dog; (2) deadlift/rowing; (3) single-leg stance and (4) side planks. Two of the exercises, bird dog and side plank, are dedicated to directly training the core-stabilizing and/or core-surrounding muscles while the other two exercises, deadlift/rowing and single-leg standing balance, are considered to impact the core indirectly by means of the upper and/or lower extremities. To account for possible side effects, side plank and single-leg stance are always performed at ten repetitions each side.

**Table 2:** Exercise details: for each exercise, level (1–3), surface (stable/unstable) and description including perturbation are provided

	Exercise 1: bird dog		Exercise 2: deadlift/rowing		Exercise 3: single-leg standing balance		Exercise 4: side planks	
					
Exercise Level	Task	Perturbation	Task	Perturbation	Task	Perturbation	Task	Perturbation
Level 1 (1st week)	Hand and knee stance diagonal arm and leg: from body center upwards (horizontal) on unstable surface	Terra Band Instability	Rowing in ball stance on stable surface	Terra Band	Unipedal stance plus hip abduction on stable surface	Terra Band	Knee on ground; hip up/down	Instability
Level 2 (2nd week)	Hand and feet stance: bending, stretching a leg on stable surface	Terra Band	Rowing in ball stance on unstable surface	Terra Band Instability	Unipedal stance plus hip abduction and leg extension on stable surface	Terra Band	Knee on ground; hip up/down on unstable surface	Ball task (roll ball to the wall and catch it back)
Level 3 (3rd week)	Hand and feet stance: bending, stretching a leg on unstable surface	Terra Band Instability	One-handed rowing on stable surface	Terra Band	Unipedal stance plus hip abduction and leg extension on unstable surface	Terra Band Instability	Legs stretched, hip fixed upwards on stable surface	Ball task (roll from hand to feet and vice versa)

Intervention group I (G I) performs three exercises (rowing, side plank, bird dog) with two sets of 10 repetitions for each exercise, with a minimum of 30 s rest in between sets. In between exercises, again a 2-min break is held. Intervention group II (G II) performs two exercises (bird dog, side plank) with two sets of 10 repetitions for each exercise and a 2-min break in between exercises. The training program of intervention group III (G III) consists of one exercise (side plank) with three sets of 10 repetitions (30 s rest in between sets).

In all intervention groups, all exercises comprise three different levels. To enhance the efficacy of the exercises, perturbations are integrated into all exercises in all three intervention groups with the aim of increasing the difficulty and, therefore, the demands on the trunk-muscle activity. Details of the four different exercises and the levels including perturbation are presented in Table 2.

#### Therapy monitoring

During the (home-based) intervention phase, a standardized training log is completed by each participant documenting each training session. The progression of the 3-week intervention is explained and demonstrated during a guided training session prior to the start of the home-based intervention.

### Randomization procedure and blinding

After inclusion, participants are randomized into either one of the three interventions or the control group by the study coordinator. The randomization order follows the study inclusion order. The randomization list is generated using a computer-based algorithm (www.randomization.com). All investigators (measurement team) other than the study coordinator and the therapist are blinded. Participants are instructed not to communicate their group allocation to other participants or study staff.

### Experimental procedure

**Fig. 3:**
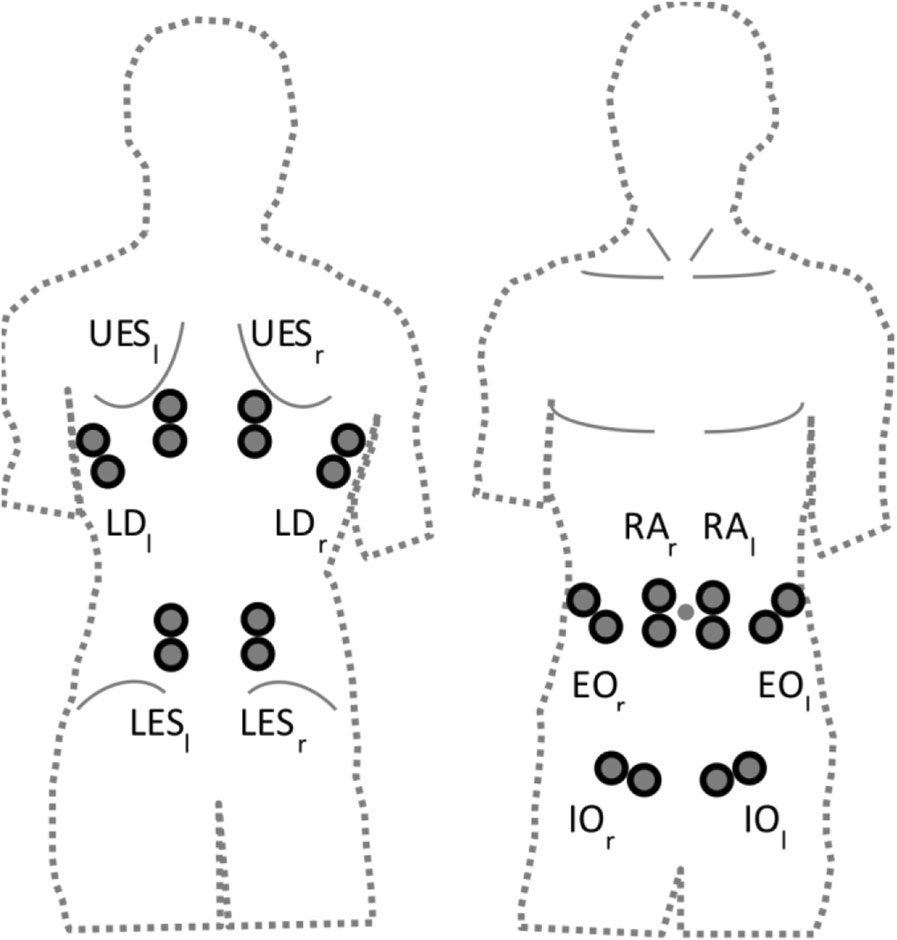
Twelve-lead surface electromyographic (EMG) – trunk setup: RA_ri/le_ = M. rec. abd. right/left, EO_ri/le_ = M. obl. ext. abd. right/left, IO_ri/le_ = M. obl. int. abd. right/left; LD_ri/le_ = M. latis. dorsi right/left, UES_ri/le_ = M. erec. spinae thoracic (T9) right/left, LES_ri/le_ = M. erec. spinae lumbar (L3) right/left

**Fig. 4:**
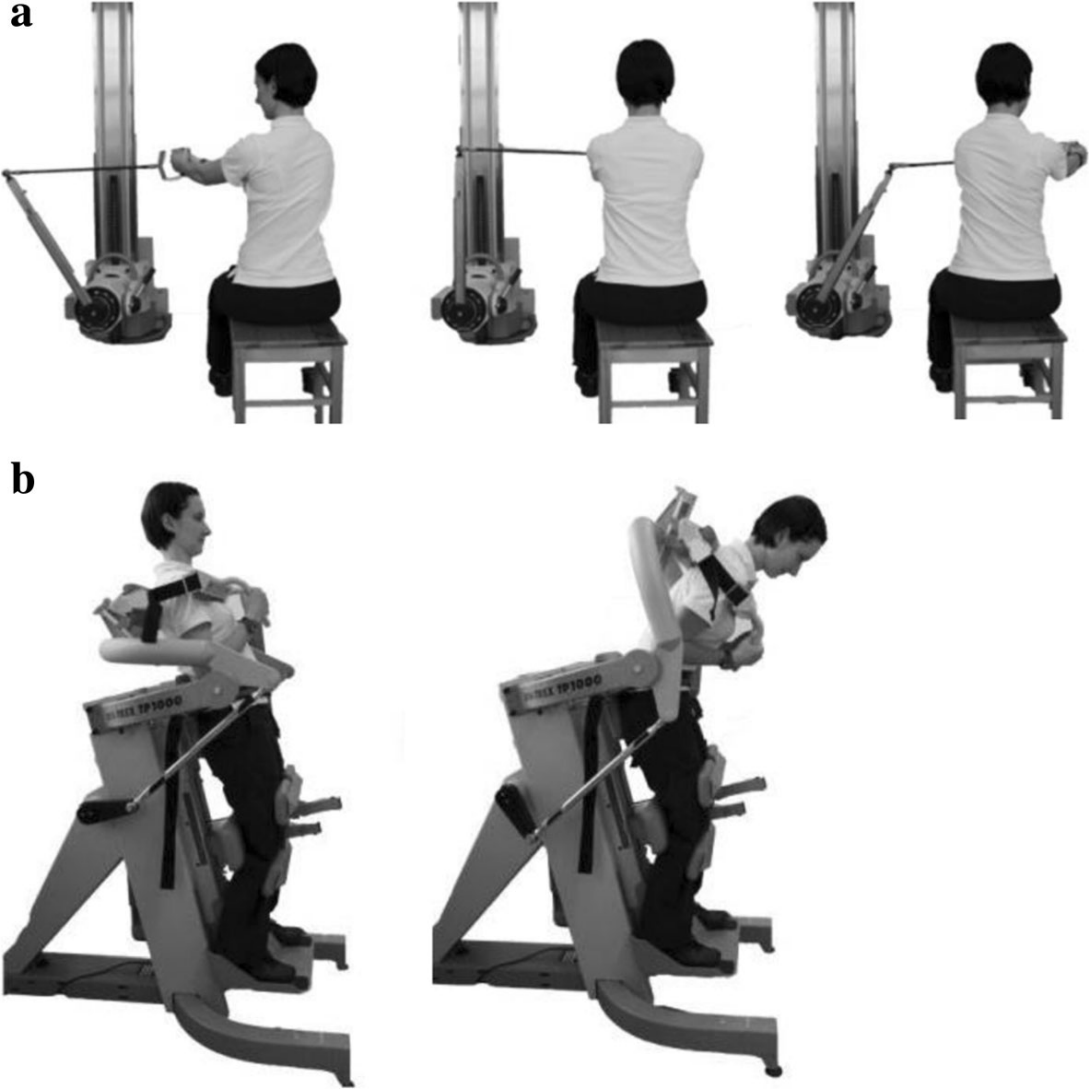
Assessment of trunk strength and sudden trunk loading. **a** trunk rotation (left/right); 31.5° left rotation to 31.5° right rotation (CON-TREX WS; Physiomed Elektromedizin, Germany). **b** trunk extension and flexion; 10° extension (left) to 45° flexion (right) (CON-TREX MJ/TP 1000; Physiomed Elektromedizin, Germany)

A test-retest design with two measurement days and a 3-week, home-based, core-specific sensorimotor intervention is performed after the randomization procedure. The control group performs a standard intervention program as well. All test procedures are performed identically on both measurement days (M1 = prior; M2 = post intervention) in a standardized order. After assessing the anthropometrics (height, weight), a medical check-up is conducted to ensure that all participants are suitable for the upcoming trunk-strength testing. Participants are then prepared for surface electromyographic measurements (EMG) of the trunk. Twelve pairs of EMG electrodes are positioned over the ventral and dorsal muscles (Fig. 3; [4]). All participants undergo a general physical warm-up of at least 5 min prior to testing. Subsequently, a trunk-strength protocol, including isometric maximum voluntary contraction (MVC), isokinetic and sudden trunk loading for rotation as well as flexion and extension testing, is applied by an isokinetic dynamometer (CON-TREX MJ/TP, Physiomed AG, Schnaittach, Germany). The MVC measurement is used as a reference for the EMG-normalization procedure (Fig. 4). Subjective rating of load-induced back pain is assessed directly before and after the trunk-loading protocol (Visual Analog Scale VAS: 0.0–10.0 cm) to control for possible pain effects [13, 14].

### Outcome measures

#### Trunk strength

The protocol consists of isokinetic strength testing of (right-sided) rotation as well as flexion/extension in a randomized order. Only right-sided rotation is tested due to the assumption of non-existing differences between sides in the cohort to be analyzed, as reported by Lindsay and Horton [15]. Trunk-strength rotation is measured in a seated position with a range of motion from 31.5° left-side to 31.5° right-side rotation (ROM 63°) using an angular dynamometer (CON-TREX WS, Physiomed Elektromedizin AG, Schnaittach, Germany) (Fig. 4a). Trunk flexion and extension is performed in a standing position using a second dynamometer (CON-TREX MJ/TP 1000, Physiomed Elektromedizin AG, Germany) (Fig. 4b). Participants are fixed to the dynamometer by adjustable adapters at the lower leg and knee, as well as by two non-stretching belts at the hip and upper body [16, 17]. Total range of motion is set at 55°, from 10° extension to 45° flexion.

Both conditions begin with an additional 60-s period of specific warm-up exercises and familiarization prior to testing. Maximum strength in rotation and in flexion/extension is tested in isometric (rotation: 0°; extension/flexion 17.5°), concentric (30°/s, con) and eccentric (30°/s, ecc) modes, performing five repetitions (5 s isometric) each. Furthermore, sudden dynamic loading is induced during eccentric mode (30°/s) by a novel, customized perturbation (acceleration from 30°/s to 330°/s within 120 ms for trunk rotation and from 30°/s to 150°/s within 250 ms for trunk extension). Verbal encouragement was given throughout the entire test to ensure participants’ maximum effort. The primary outcome of the study is trunk strength (peak torque) analyzed for all test modes including peak torque (Nm) in trunk rotation, trunk flexion and trunk extension, calculated as the mean of the three highest peak torques out of five repetitions [16, 17]. In previous studies, high relative (intraclass correlation coefficient (ICC)) and absolute (TRV and Bland and Altman bias and limits of agreement) reproducibility could be shown for isokinetic trunk peak torque and response to sudden, dynamic trunk-loading assessment (peak torque with perturbation: ICC 0.94, test-retest variability 8.53 ± 6.33%; bias ± 1.96 SD: 8.16 ± 64.8 Nm; *n* = 10) [18–22].

#### Electromyography

Muscular activity of the trunk is assessed by means of a 12-lead surface EMG during all strength measurements. Analysis included six ventral (Mm rec. abd. (RA), obl. ext. abd. (EO), obl. int. abd (IO) of left and right sides) and six dorsal (Mm erec. spinae thoracic (T9; UES)/lumbar (L3; LES), latis. dorsi (LD) of left and right sides) muscles (Fig. 3) [4]. Muscular activity will be analyzed using bipolar surface EMG (band-pass filter: 5 Hz to 500 Hz, gain: 5.0, overall gain: 2500, sampling frequency: 4000 Hz; RFTD-32, myon AG, Baar, Schweiz). Localization of electrodes is carefully determined following Radebold et al. (2000). Before electrodes (AMBU Medicotest, Denmark, Type N-00-S, inter-electrode distance: 2 cm) are applied, the skin is shaved and slightly exfoliated to remove surface epithelial layers and finally disinfected. In addition, skin resistance is measured (< 5 kΩ). The longitudinal axes of the electrodes are in line with the presumed direction of the underlying muscle fibers.

The signal will be rectified and averaged before calculating outcome measures. EMG amplitudes (RMS (%)) are normalized to isometric maximum voluntary contraction (MVC). Neuromuscular activity serves as secondary outcome: MVC-normalized EMG amplitudes (EMG-RMS (%)) are calculated for each test condition and all single muscles. Furthermore, the mean EMG-RMS is calculated for four areas of the trunk (right and left ventral area: grouping of RA, EO, IO on right side and grouping of RA, EO, IO on left side, right and left dorsal area: grouping of LD, UES, LES on the right side and grouping of LD, UES, LES on the left side) [4]. Within the time domain, the onset of muscular activity (ms) will be detected and measured for the first active ventral and dorsal muscles, representing a response to the perturbation. In addition, the specific muscles (first on) for the ventral and dorsal muscles are documented.

### Statistical analysis

A power analysis to calculate sample size was not performed. The number of subjects to be included was based on previous published studies [13, 14]. *N* = 20 subjects per group will be included.

Data is collected and analyzed after the assessment is completed. After the plausibility check, the data is descriptively analyzed, including the means and standard deviations (SD) of torque (primary) and EMG (secondary) outcomes. In addition, a gain score analysis (change score analysis) using the mean difference between M1 and M2 for between-group differences of the outcomes will be performed (one-way analysis of variance (ANOVA)).

All statistical procedures are executed after the examination of underlying assumptions for parametric and non-parametric hypothesis testing. Hypotheses testing for all outcomes are performed using particular variance analytical testing (e.g., two-way ANOVA with dependent samples). The level of significance is set at *α* = 0.05 for all statistical analyses. Multiple testing will be controlled via Bonferroni adjustment.

## Discussion

The main purpose of this study is to analyze the dose-response relationship between core-specific sensorimotor intervention programs of different training intensities and trunk function, represented by maximum strength capacity, sudden loading compensation and neuromuscular activity. Therefore, a feasible study protocol based on a single-blind randomized controlled design is used.

All training programs, control and all intervention groups, consist of core-specific sensorimotor exercises [10, 12, 23]. Moreover, additive sensorimotor perturbation will be applied to one (control group) and all exercises (all intervention groups) to enhance the efficacy of neuromuscular activity while exercising [24–26]. In a pilot study, Mueller et al. [25] showed that the use of additional perturbations during core-specific sensorimotor exercises was superior for enhancing trunk neuromuscular activity. Higher EMG amplitudes might indicate higher exercise intensity, but additional functional benefits and long-term effects still need to be investigated. Although convincing evidence exists for the relevance of sensorimotor exercises and their combination with additional perturbations to enhance core stability and trunk function, fundamental information on the dose-response relationship is rather sparse.

A main strength of the planned intervention programs is the overall short exercise duration as well as the low usage of resources in the home-based intervention [10]. This cost-effective intervention might be feasible for the transfer to the general population and/or elite athletes.

One main limitation in the trial presented here is the blinding of the participants. To keep the risk of bias to a minimum, all involved investigators are blinded to the group allocation.

In summary, the presented trial provides further insight into the dose-response relationship for core-specific sensorimotor exercises and the definition of a minimum dosage required to improve trunk function. In addition, the efficacy or non-efficacy of the sensorimotor exercises targeting neuromuscular factors like strength and neuromuscular activity will be evaluated in detail.

### Trial status

German Clinical Trials, ID: DRKS00012917. Registered on 22 August 2017. Participant recruiting starts on 1 September 2017. At the time of manuscript submission, no participant is included into the study. Recruiting completion is anticipated to be May 2018, measurement completion June 2018 and study completion is expected to be July 2018.

## Additional file

Additional file 1: Standard Protocol Items: Recommendations for Interventional Trials (SPIRIT) Checklist. (DOC 122 kb)

## Abbreviations

C: Control group
DL: Left dorsal area
DR: Right dorsal area
EMG: Electromyography
EO: Musculus obl. ext. abd.
G I-III: Intervention groups I, II, III
IO: Musculus obl. int. abd.
LD: Musculus latissimus dorsi
LES: Musculus erec. spinae lumbar (L3)
MVC: Maximum voluntary contraction
RA: Musculus rec. abd.
SMT: Sensorimotor training
UES: Musculus erec. spinae thoracic (T9)
VL: Left ventral area
VR: Right ventral area
